# Neuroprotective Effects of Novel Treatments on Acute Optic Neuritis—A Meta-Analysis

**DOI:** 10.3390/biomedicines10010192

**Published:** 2022-01-17

**Authors:** Tsung-Hsien Tsai, Chao-Wen Lin, Li-Wei Chan, Teck-Boon Tew, Ta-Ching Chen

**Affiliations:** 1 Department of Medical Education, National Taiwan University Hospital, Taipei 100, Taiwan; thtsai@ntuh.gov.tw; 2 Department of Ophthalmology, National Taiwan University Hospital, Taipei 100, Taiwan; wesley.lin1@gmail.com; 3 Department of Ophthalmology, Taipei Tzu Chi Hospital, The Buddhist Tzu Chi Medical Foundation, New Taipei City 231, Taiwan; ichanlw@gmail.com; 4 Department of Ophthalmology, National Taiwan University Hospital Hsin-Chu Branch, HsinChu County 302, Taiwan; b94401119@ntu.edu.tw

**Keywords:** optic neuritis, neuroprotection, optical coherence tomography, retinal nerve fiber layer, ganglion cell and inner plexiform layer, corticosteroid

## Abstract

Optic neuritis, inflammation of the optic nerve, can cause visual impairment through retinal nerve fiber layer (RNFL) degeneration. Optical coherence tomography could serve as a sensitive noninvasive tool for measuring RNFL thickness and evaluating the neuroprotective effects of treatment. We conducted a meta-analysis to compare RNFL loss between novel add-on treatments and corticosteroid therapy at least 3 months after acute optic neuritis. The outcome measures were mean differences (MDs) in (1) RNFL thickness compared with the baseline in the affected and unaffected eye and (2) LogMAR visual acuity (VA). Seven studies involving five novel agents (memantine, erythropoietin, interferon-beta, phenytoin, and clemastine) were analyzed. When compared with the baseline RNFL thickness of the affected eye, the neuroprotective effects of novel add-on treatments could not be demonstrated. The difference in visual outcomes was also not significant between the two treatment groups. One study revealed that phenytoin has the potential to alleviate RNFL loss when the baseline thickness of the unaffected eye is considered. Larger randomized controlled trials with suitable outcome measures are warranted to evaluate the neuroprotective effects of novel treatments. Further studies should also tailor therapies to specific patient populations and investigate a more targeted treatment for acute optic neuritis.

## 1. Introduction

Optic neuritis, which can be clinically divided into typical and atypical forms, refers to inflammation of the optic nerve that can cause vision impairment through the degeneration of the optic nerve and retinal nerve fibers. Typical optic neuritis is a demyelinating disorder of the optic nerve that is often associated with multiple sclerosis (MS), whereas atypical optic neuritis involves inflammatory, infectious, or autoimmune etiologies [1,2]. The two cardinal symptoms of typical optic neuritis are unilateral vision loss and painful eye movement. Visual loss develops in hours or days, and the onset of eye pain usually coincides with it. Other symptoms include visual field (VF) loss, dyschromatopsia, and decreased contrast sensitivity. Recovery often starts within a month, and prognosis is typically favorable [3]. By contrast, atypical optic neuritis generally occurs bilaterally, and its clinical manifestations are more severe. Certain features, such as pronounced disc swelling, peripapillary hemorrhages, and macular exudates, are indicative of atypical optic neuritis. Visual impairment may become profound and not improve [1,2].

The treatment of acute optic neuritis is critical because persistent visual impairment, including the disturbance of a patient’s visual acuity (VA), VF, and contrast sensitivity, can seriously affect their quality of life. One study reported that 59% of patients with untreated typical optic neuritis experienced visual disturbances within a year [4]. In 1992, a large-scale randomized trial (Optic Neuritis Treatment Trial) established treatment guidelines for acute optic neuritis [5]. To date, the mainstay of treatment has been intravenous corticosteroid. Although steroid treatment can hasten recovery of symptoms in the short term, it does not influence long-term visual outcomes or atrophy of the optic nerve [6].

Visual outcomes such as VA and VF are the most common measures for evaluating treatment responses in patients with acute optic neuritis. However, these measures are subjective and inconsistent and are affected by the ceiling effect. Technological advances have provided several quantitative measures for optic nerve pathologies, including retinal nerve fiber layer (RNFL) thickness and ganglion cell–inner plexiform layer (GCIPL) thickness, which can be investigated using optical coherence tomography (OCT). Previous studies have revealed that RNFL thinning is correlated with poor visual outcomes in patients with acute optic neuritis and with disability in patients with MS [7,8,9]. The assessment of RNFL thickness could allow direct observation of neuroaxonal damage, indicating RNFL thickness to be a marker of axonal loss [10]. A longitudinal in vivo study also demonstrated that GCIPL thinning after acute optic neuritis may be a derivative of optic nerve pathology [11]. Reduced ganglion cell layer thickness can be detected earlier than reduced RNFL thickness [12]. RNFL thickness and GCIPL thickness have been demonstrated to be useful primary outcome parameters for determining the neuroprotective effects of treatment in optic neuritis trials [13,14,15].

The aim of this meta-analysis was to investigate the neuroprotective effects of all potential treatments for acute optic neuritis on the basis of RNFL or GCIPL thickness, measured using OCT. We conducted this study to determine whether novel treatments for acute optic neuritis can reduce axonal loss and RNFL thinning compared with standard methylprednisolone treatment.

## 2. Materials and Methods

### 2.1. Eligibility Criteria for Selecting Studies

We included randomized controlled trials (RCTs), cohort studies, and case–control studies that reported acute optic neuritis treatments and OCT findings of patients at baseline and after treatment. Studies targeting patients with acute optic neuritis were included. Inclusion criteria included being at least 18 years old with symptoms appearing <30 days prior to seeing a doctor or being recruited. The included studies had to be comparative studies and had to clearly specify their treatment regimens as well as routes and dosages, with the control arm using the current standard treatment, intravenous methylprednisolone for 3–5 days followed by oral prednisolone. For OCT outcome assessment, we included studies examining RNFL or GCIPL thickness at baseline and at least 3 months after the onset of acute optic neuritis to calculate the mean difference between pretreatment and posttreatment values.

We excluded studies that did not clearly report the duration of symptom onset or the route and dosage of treatment. We also excluded studies involving patients with major comorbid ocular diseases or other systemic diseases.

### 2.2. Search Methods

We conducted a systematic search of Pubmed and Embase for studies written in any language and published between 1 January 1991, and 18 August 2021. The following search keywords were used: [optic neuritis] AND [optical coherence tomography OR retinal nerve fiber layer OR ganglion cell inner plexiform layer]. The detailed search strategy is outlined in Appendix A. We also reviewed the references of all included articles to identify potentially relevant studies.

### 2.3. Data Screening, Data Extraction, and Risk of Bias Assessment

Search results from PubMed and Embase were imported into Endnote 20 software (Microsoft, Washington, DC, USA), which was used to identify and remove any duplicate articles. Two investigators (T.-H.T. and C.-W.L.) independently reviewed the titles and abstracts of all studies. The full texts of all potentially eligible articles were then evaluated according to the inclusion and exclusion criteria. Any disagreements were resolved through discussion with a third investigator (T.-C.C.).

Subsequently, we collected the following information from each eligible study: (1) general study characteristics, namely author name, year of publication, title of study, journal name, study design, study population, number of patients, and duration from symptom onset; (2) treatment variables, namely treatment regimen (type and duration) and treatment dosage and route; and (3) outcome measures, namely time of outcome measurement, mean thickness of the peripapillary RNFL or macular GCIPL, and VA record (LogMAR), if available.

The quality of eligible articles was assessed by two investigators (T.-H.T. and C.-W.L.) independently. The Cochrane risk of bias tool was used for RCTs, and the Newcastle–Ottawa Scale was used to assess cohort studies.

### 2.4. Data Synthesis and Analysis

The results from the selected articles were combined for statistical analysis. OCT findings (RNFL or GCIPL thickness) and LogMAR VA were calculated as continuous variables, and the mean pretreatment and posttreatment thickness differences with standard deviations (SDs) were calculated for each study for pooling analysis. If a study did not provide this information, we derived the SD from the pretreatment and posttreatment SDs in the study by using a paired t test with a correlation coefficient of 0.5. A random-effects model analysis was performed for all outcomes, and heterogeneity across studies was evaluated using I^2^ statistics. All analyses were performed using Review Manager 5.3 (The Cochrane Collaboration, Oxford, England).

## 3. Results

### 3.1. Study Characteristics

**Figure 1:**
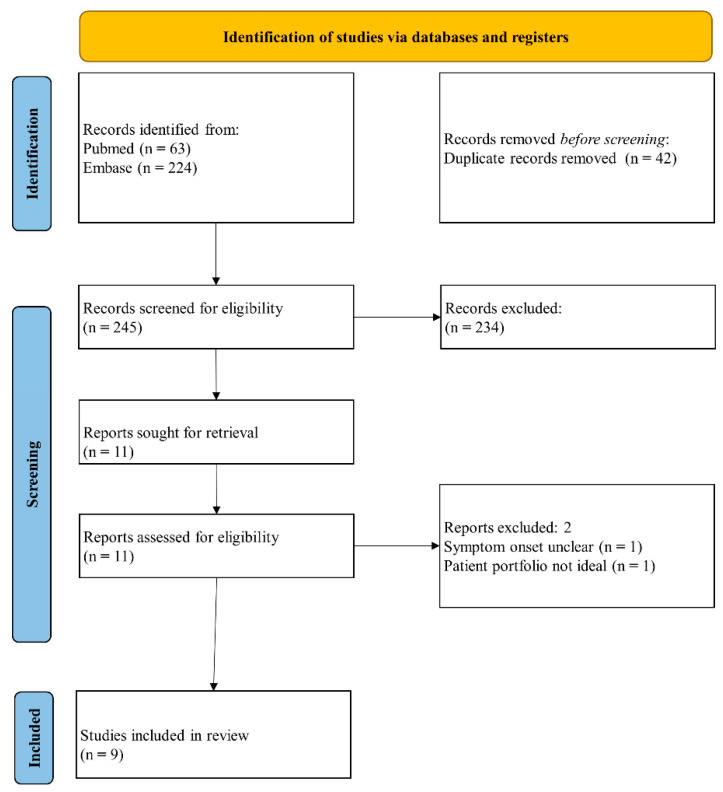
Flow diagram of the study selection process.

Our search of Pubmed and Embase retrieved 63 and 224 articles, respectively. After removing 42 duplicates, we screened the titles and abstracts of 245 studies, among which 11 articles met our inclusion criteria. We performed a full-text evaluation of these articles, and two articles were further excluded: one because of unclear duration from symptom onset [16] and the other because it targeted patients with vitamin D deficiency, which was not the focus of our study [17]. Ultimately, nine studies were eligible for inclusion in our meta-analysis [13,14,15,18,19,20,21,22,23]. The study selection process is illustrated in Figure 1.

The characteristics of the nine studies are summarized in Table 1. In addition to corticosteroids, these studies covered seven novel agents for acute optic neuritis, namely amiloride, atacicept, clemastine, erythropoietin (EPO), interferon (INF)-beta, memantine, and phenytoin. Among the included studies, two were prospective cohort studies, and the others were RCTs. Regarding study population, one study specifically investigated acute optic neuritis in patients with MS [21], and two studies recruited patients with acute optic neuritis as a clinically isolated syndrome [19,22]. Although we considered all studies with a duration of symptom onset within 30 days, only three studies included patients who had symptoms for more than 14 days prior to seeking medical care or being recruited. We removed the studies of McKee et al. and Sergott et al. from our final analysis to minimize biases because instead of treating every patient with optic neuritis by administering a standard dose of methylprednisolone, the researchers optionally administered a steroid to their control groups [22,23]. With the exception of these two trials, all patients from other studies received at least intravenous methylprednisolone 1000 mg for 3–5 days as the standard treatment.

In terms of OCT outcome, three studies evaluated follow-up OCT at 6 months [14,15,20], two studies at 4 months [13,19], and two studies at 3 months [18,21]. All seven studies collected RNFL thickness as their outcome; two of these studies provided data on RNFL thickness in the unaffected fellow eye at baseline [15,19]. The RNFL thickness of the unaffected eye could be used as an alternative because the disc and nerve fibers of the affected eye are typically swollen in the acute phase of optic neuritis. We performed meta-analyses to compare the change in RNFL thickness with that of the baseline for the affected eye as well as the unaffected fellow eye. In addition to RNFL thickness, Moghaddasi et al. included ganglion cell layer thickness as one of their outcomes [21]. Yadegari et al. selected GCIPL thickness as their primary outcome of interest [14]. Because of limited data on ganglion cell layer or GCIPL thickness in our included studies, our meta-analysis was performed using RNFL data only.

### 3.2. Risk of Bias

**Figure 2:**
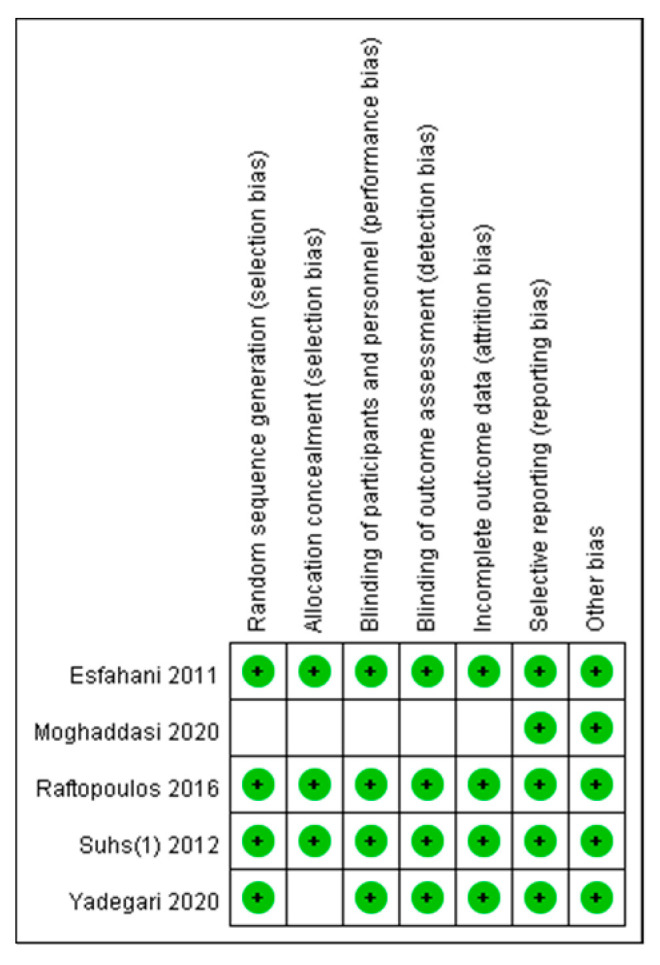
Methodological quality of included randomized controlled trials.

The methodological quality of the included RCTs is summarized in Figure 2. Five trials were described as RCTs, each reporting an acceptable method of randomization [13,14,15,18,21]. All RCTs described the generation of random sequences with computer software or number tables and emphasized allocation concealment during randomization, except for the studies of Moghaddasi et al. [21] and Yadegari et al. [14]. Four trials conducted double-blinding of the participants, personnel, and outcome assessors, but the study of Moghaddasi et al. [21] lacked an explanation of their blinding procedure. Double-blinding of the participants and personnel was achieved by providing drugs and placebos with an identical appearance. Only the study of Moghaddasi et al. was ranked as unknown risk because the number of patients lost to follow-up was not mentioned [21]. All studies were ranked as low risk in terms of reporting bias and other bias. The two cohort studies had a score of 9 on the Newcastle–Ottawa scale, demonstrating a low risk of bias from the studies [19,20].

### 3.3. Mean Differences in RNFL Thickness Compared with Baseline Data of the Affected Eye

**Figure 3:**
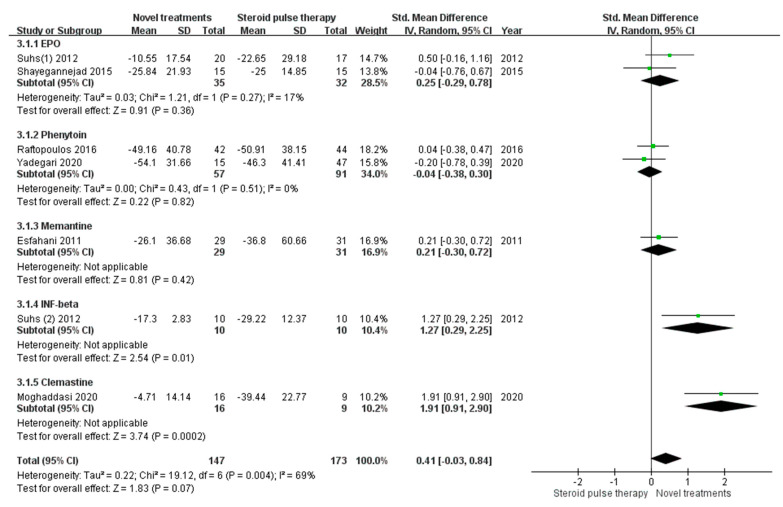
Mean differences in retinal nerve fiber layer thickness compared with baseline data of the affected eye. (CI, confidence interval; IV, inverse variance; SD, standard deviation; EPO, erythropoietin; INF, interferon).

We compared RNFL thickness at follow-up with the baseline RNFL thickness of the affected eye (Figure 3). Among the seven studies, two studies examined EPO in their treatment groups, both of which used the same dosage [13,20], and two studies investigated phenytoin with similar regimens [14,15]. In the EPO group, the pooled mean difference in RNFL thickness was 0.25 (95% CI −0.27 to 0.78, test for overall effect: Z = 0.94, *p* = 0.34), with nonsignificant heterogeneity (I^2^ = 19%, Q-test *p* = 0.27). In the phenytoin group, the pooled mean difference in RNFL thickness was −0.04 (95% CI −0.38 to 0.30, test for overall effect: Z = 0.22, *p* = 0.82), and no heterogeneity was observed (I^2^ = 0%, Q-test *p* = 0.51).

When we pooled the data from different treatments, the mean difference in RNFL thickness did not differ between the novel treatment and standard treatment groups, with the overall mean difference of RNFL thickness being 0.41 (95% confidence interval [CI] −0.03 to 0.84, test for overall effect: Z = 1.84, *p* = 0.07). The I^2^ value from the analysis was 69%, with a Q-test *p* value of 0.004, indicating significant heterogeneity.

### 3.4. Mean Differences in RNFL Thickness Compared with Baseline Data of the Unaffected Eye

**Figure 4:**
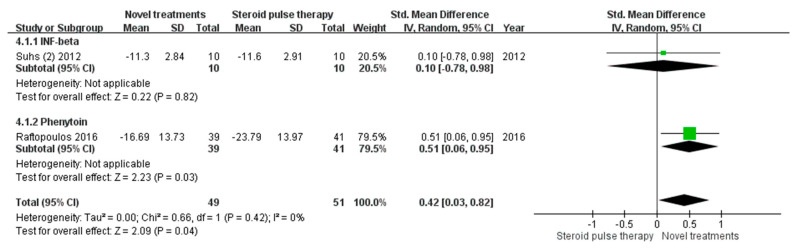
Mean differences in retinal nerve fiber layer thickness compared with baseline data of the unaffected eye. (CI, confidence interval; IV, inverse variance; SD, standard deviation; INF, interferon).

We also compared the RNFL thickness at follow-up with the baseline RNFL thickness of the unaffected fellow eye (Figure 4). Only two studies provided baseline data on RNFL thickness of the unaffected eye [15,19]. The mean difference in RNFL thickness was larger in the standard treatment group than in the novel treatment group. The overall mean difference in RNFL thickness was 0.42 (95% CI 0.03 to 0.82, test for overall effect: Z = 2.09, *p* = 0.04). The I^2^ value from the analysis was 0% (Q-test *p* = 0.42), indicating no heterogeneity. However, the medications used in these two studies were different and one study [15] represented about 80% of effect size.

### 3.5. Mean Difference in LogMAR VA

**Figure 5:**
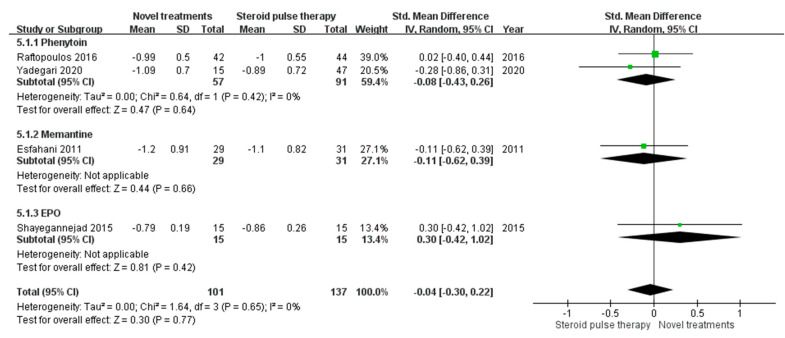
Mean differences in LogMAR visual acuity. (CI, confidence interval; IV, inverse variance; SD, standard deviation; EPO, erythropoietin).

In addition to OCT outcomes, we also performed a meta-analysis to compare visual outcomes (LogMAR VA) (Figure 5). Among all seven studies, only four studies [14,15,18,20] provided data on LogMAR VA and two studies [14,15] used phenytoin as the treatment. In the phenytoin group, the mean difference in LogMAR VA was −0.08 (95% CI −0.43 to 0.26, test for overall effect: Z = 0.47, *p* = 0.64), and no heterogeneity was observed (I2 = 0%, Q-test *p* = 0.42). When we pooled the data from different treatments, the mean difference in LogMAR VA did not differ between the novel treatment and standard treatment groups, with the overall mean difference being −0.04 (95% CI −0.3 to 0.22, test for overall effect: Z = 0.30, *p* = 0.77). No heterogeneity was observed (I^2^ = 0%, Q test *p* = 0.65).

## 4. Discussion

Optic neuritis, inflammation of the optic nerve, typically affects young adults between 20 and 45 years old, with a strong female predominance [24]. When a patient’s vision does not recover after an acute episode, reduced mobility may ensue, thus creating a considerable socioeconomic burden. In the past, most studies have generally focused on visual outcomes such as VA and VF when evaluating treatment responses to novel therapies; however, these measures are subjective and relatively crude. Novel visual outcome measures, such as low-contrast visual acuity (LCVA), can provide highly accurate quantification of functionally relevant visual deficits [25]. However, these measures are still not available in general practice. Inflammation of the optic nerve can lead to retrograde degeneration of the RNFL, a pure compartment of unmyelinated axons [9]. OCT is a noninvasive technique for obtaining detailed images of all retinal layers at high resolution; the degree of axonal damage to the RNFL can be measured to within 1 micron [26]. OCT provides an objective, reproducible, and accurate measure for directly evaluating the potential neuroprotective effects of novel treatments.

Among the included studies in our meta-analysis, we evaluated five novel agents for treating acute optic neuritis, namely memantine, EPO, INF-beta, phenytoin, and clemastine. In each study, the potential add-on effects of the novel treatment were compared with the standard treatment, intravenous corticosteroid. The potential mechanisms by which these treatments achieve neuroprotection differ. Memantine is an N-methyl-D-aspartate (NMDA) receptor antagonist. The neuroprotective effect of memantine in neurodegenerative diseases, such as Alzheimer disease, has been demonstrated [27]. EPO exhibits neurotrophin-like properties, and axon protection is most effective when EPO is combined with high-dose methylprednisolone, as observed in a rat model of myelin oligodendrocyte glycoprotein (MOG)-induced experimental autoimmune encephalomyelitis [28]. IFN-beta is a type of disease-modifying therapy for MS and can delay the conversion of clinically isolated syndrome (CIS) into clinically definite MS [29]. Phenytoin, an anticonvulsant, is a selective sodium-channel inhibitor, and its neuroprotective effects have been proven in experimental models [30]. Clemastine is a first-generation antihistamine that can enhance the remyelination of optic nerves [31]. Early administration of intravenous corticosteroid in acute optic neuritis could suppress inflammation but may not prevent neuronal loss and RNFL thinning [32]. Instead, steroid treatment may even increase retinal ganglion cell degeneration through the blocking of neurotrophin-dependent pathways, as demonstrated in an animal model of optic neuritis [33].

Our meta-analysis compared the effects of intravenous corticosteroid treatment and novel add-on treatments on RNFL thickness before and after therapy. When we chose the RNFL thickness of the affected eye as the baseline, there was no significant neuroprotective effect in the EPO and phenytoin treatment groups. The two studies involving EPO had different designs, with an I^2^ of 19%. Suhs et al. conducted an RCT with a follow-up period of 16 weeks and showed that RNFL thinning was less apparent after EPO treatment [13]; however, Shayegannejad et al. identified no significant difference between EPO and intravenous methylprednisolone treatment in a cohort study with a follow-up period of 6 months [20]. The overall effect of EPO treatment revealed no significant difference in neuroprotection compared with conventional steroid treatment. Further studies are required to ascertain a suitable follow-up period for determining the effect of EPO treatment on patients with acute optic neuritis. The two studies investigating phenytoin were both RCTs, with similar treatment and control groups [14,15]. However, the overall effect was also not significant, with an I^2^ of 0%. When we pooled the data from different novel treatment options, RNFL thinning tended to be less apparent in the novel treatment group, but the difference was not statistically significant. Besides, a high level of heterogeneity was observed across these studies.

Alternatively, when we chose the RNFL thickness of the unaffected fellow eye as the baseline, the neuroprotective effects of the novel treatments could be more evident. Only two studies provided baseline data on RNFL thickness of the unaffected eye [15,19], and the treatment options in these two studies were different. The major difference was observed in the study of Raftopoulos et al. [15]. In their study, the difference between the two treatment groups compared with the RNFL thickness of the affected eye at baseline was not significant. However, they compared the 6-month RNFL thickness of the affected eye with the baseline data of the unaffected eye. The phenytoin group was observed to have significantly less RNFL thinning. The RNFL thickness of the affected eye is significantly increased at presentation, and the resolution of disc edema may require a mean of 1.6 months [34]. The variation of RNFL swelling in the affected eye at baseline could be a confounder. The use of RNFL thickness in the unaffected eye at presentation for comparisons may be more appropriate because the RNFL should be comparable in both eyes in patients without previous insults. Further randomized controlled trials with suitable outcome measures are warranted to evaluate the neuroprotective effects of novel treatments.

Two of the five novel treatments exerted significant neuroprotective effects, namely INF-beta and clemastine fumarate. INF-beta therapy is the established treatment option for MS, and clemastine fumarate has been demonstrated in an RCT to be a remyelinating therapy for MS [31]. For both treatments, each study enrolled specific groups of patients. In the INF-beta study, Suhs et al. included patients with CIS, which is generally regarded as the first manifestation of MS [19]; however, in the clemastine study, Moghaddasi et al. enrolled only patients with MS [21]. This observation could indicate that treatments for acute optic neuritis should be tailored to different etiologies, such as MS, neuromyelitis optica (NMO), or MOG-associated disease.

We also investigated visual outcomes in the included studies of our meta-analysis. Only four studies provided pretreatment and posttreatment LogMAR VA data [14,15,18,20]. However, the difference in visual outcomes between novel and standard treatments was not significant in all studies. The overall effect of the meta-analysis was also not significant, with an I^2^ of 0%. RNFL thickness could be used to predict visual recovery after optic neuritis, especially the impairment of VF [8,35]. Some studies reported that thinning of RNFL is correlated with VA in patients with MS, but the correlation is higher between RNFL thickness and LCVA [36,37]. As mentioned, VA is a crude measure, and small effects may not be detected. Moreover, VA depends on only a small area of the fovea, which comprises minimal RNFL. For trials investigating acute optic neuritis, promising outcome measures could include RNFL and GCIPL thickness measured by OCT, as well as functional outcomes such as VF, LCVA and VA [25].

This study had several limitations. First, considerable heterogeneity was observed, which may have resulted from differences in study designs, OCT machines, patient populations, and mechanisms of action of the included treatments. Similar to a previous meta-analysis, we pooled estimated differences of OCT data among studies to minimize interstudy variations [38]. Second, the target study population in the analyzed studies was mostly patients with typical optic neuritis, such as in MS or CIS. The results may be different in optic neuritis with positive serum aquaporin−4 or MOG antibodies. Third, most of the included studies used the RNFL thickness of the affected eye as the baseline RNFL thickness. Swollen nerve fibers in the acute phase may have influenced the analysis, as swollen fibers would distort the original nerve fiber thickness. Finally, the sample sizes of the included studies were small, and only a few RCTs have been conducted on this topic.

In conclusion, with advanced OCT technology, RNFL thickness has been validated as a sensitive and reproducible measure for evaluating the neuroprotective effects of treatment. For patients with acute optic neuritis, the neuroprotection of novel add-on treatments such as EPO and phenytoin could not be demonstrated when compared with the baseline RNFL thickness of the affected eye. One study showed that phenytoin holds the potential to alleviate RNFL loss compared with standard intravenous corticosteroid treatment when considering baseline data of the unaffected eye. In the future, large well-designed clinical trials with appropriate outcome measures are required to investigate the neuroprotective effects of novel treatments. Furthermore, studies should tailor therapies to specific patient populations, such as those with MS, NMO, or MOG-associated disease, to investigate a more targeted treatment for acute optic neuritis.

## Figures and Tables

**Table 1:** Characteristics of included studies.

Author (Year).	Study Design	Study Population	Number of Patients	ON Symptom Onset	Novel Treatment	Control	OCT Outcome	Time of OCT f/u
Articles included in the final analysis								
Esfahani et al. (2011) [18]	RCT	New onset unilateral ON	60	≤8 days	Memantine 5 mg for first week and 10 mg for the next 2 weeks after standard treatment	Placebo after standard treatment	pRNFL thickness	3 months
Suhs et al. (1) (2012) [13]	RCT	New onset ON	40	≤10 days	IV EPO 33,000 IU for 3 days after standard treatment	Placebo after standard treatment	RNFL thickness	4 months
Suhs et al. (2) (2012) [19]	Cohort study	ON as CIS	20	≤10 days	Subcutaneous INF-beta after standard treatment	Standard treatment	pRNFL thickness	4 months
Shayegannejad et al. (2015) [20]	Cohort study	Unilateral ON of unknown or demyelinating origin	30	≤10 days	IV EPO 33,000 IU for 3 days after standard treatment	Placebo after standard treatment	pRNFL thickness	6 months
Raftopoulos et al. (2016) [15]	RCT	Unilateral demyelinating ON	86	≤14 days	Oral phenytoin after standard treatment	Placebo after standard treatment	pRNFL thickness	6 months
Moghaddasi et al. (2020) [21]	RCT	MS with acute ON	25	≤30 days	Clemastine flumarate 1 mg twice a day for 3 months after standard treatment	Placebo after standard treatment	pRNFL and GCL complex thickness	3 months
Yadegari et al. (2020) [14]	RCT	Unilateral ON	74	≤14 days	Oral phenytoin for 3 months after standard treatment	Placebo after standard treatment	pRNFL and mGCIPL thickness	6 months
Articles not included in the final analysis								
Sergott et al. (2015) [22]	RCT	Unilateral ON as CIS	34	≤28 days	Subcutaneous atacicept for 9 months	+/−steroids	pRNFL thickness	9 months
McKee et al. (2017) [23]	RCT	New onset unilateral ON	48	≤28 days	Amiloride 10 mg daily for 3 months +/− steroids	+/−steroids	pRNFL thickness	6 months

ON, optic neuritis; OCT, optical coherence tomography; F/U, follow up; RCT, randomized controlled trial; pRNFL, peripapillary retinal nerve fiber layer; IV, intravenous; EPO, erythropoietin; CIS, clinically isolated syndrome; INF, interferon; MS, multiple sclerosis; GCL, ganglion cell layer; mGCIPL, macular ganglion cell-inner plexiform layer.

## Data Availability

All data generated or analyzed during this study are included in this published article.

## Appendix A

Embase

(‘optic neuritis’:ab,ti OR ‘optic neuritides’:ab,ti) AND (‘oct’:ab,ti OR ‘optical coherence tomography’:ab,ti OR ‘rnfl’:ab,ti OR ‘retinal nerve fiber layer’:ab,ti OR ‘gcipl’:ab,ti OR ‘ganglion cell inner plexiform layer’:ab,ti)

AND (1996:py OR 1998:py OR 1999:py OR 2002:py OR 2003:py OR 2005:py OR 2006:py OR 2007:py OR 2008:py OR 2009:py OR 2010:py OR 2011:py OR 2012:py OR 2013:py OR 2014:py OR 2015:py OR 2016:py OR 2017:py OR 2018:py OR 2019:py OR 2020:py OR 2021:py) AND (‘case report’/de OR ‘clinical trial’/de OR ‘observational study’/de OR ‘randomized controlled trial’/de OR ‘comparative study’/de OR ‘cross sectional study’/de) AND ‘article’/it

Pubmed

((“optic neuritis”[MeSH Terms]) OR (“optic neuritis”[tiab]) OR (“optic neuritides”[tiab])) AND ((OCT[tiab]) OR (optical coherence tomography[tw]) OR (RNFL[tiab]) OR (retinal nerve fiber layer[tw]) OR (GCIPL[tiab]) OR (ganglion cell inner plexiform layer[tw])) AND ((clinicaltrial[Filter] OR controlledclinicaltrial[Filter] OR observationalstudy[Filter] OR randomizedcontrolledtrial[Filter]) AND (1991/1/1:2021/8/18[pdat]))
